# Triglyceride Storage is Upregulated in Different Mouse Non-Melanoma Skin Cancer Cell Lines

**DOI:** 10.17912/micropub.biology.002182

**Published:** 2026-07-22

**Authors:** Shannon Nowotarski, Ryan Cunnane, Marissa Catteau, Justin DiAngelo

**Affiliations:** 1 Science, Penn State Berks, Reading, PA, USA

## Abstract

Links between tumorigenesis and lipid metabolism have been observed in various cancers, but whether lipid metabolism is altered in skin cancers is not well understood. Here we show that two different mouse skin cancer cell lines accumulate more triglycerides when compared to normal keratinocytes. This suggests that lipid metabolism is altered in mouse skin cancer tumor progression and may have implications for the regulation of lipid metabolism in non-melanoma skin cancer in humans.

**Figure 1. Triglyceride storage is higher in murine skin papillomas and squamous cell carcinoma cells when compared to normal keratinocytes:** Triglyceride levels were measured in murine normal keratinocytes (C5) (n=60), papilloma (P1) (n=48), or spindle cell carcinoma cells (A5) (n=60). Triglycerides were normalized to total protein. Bars indicate mean +/- standard error. *p < 0.05 when compared to C5 and £ p<0.005 when compared to C5 as determined by one-way ANOVA and post-hoc Tukey test.

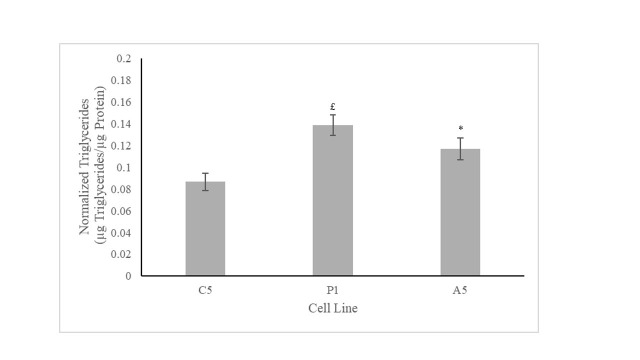



## Description

**Description**

Non-melanoma skin cancer (NMSC) is the most commonly diagnosed cancer in the United States. The two major forms of NMSC are basal cell carcinomas (BCCs) and squamous cell carcinomas (SCCs), with BCC incidence being 2-times higher than that of SCCs. BCCs are typically slow growing and rarely metastasize, whereas SCCs are often invasive and are more likely to metastasize (Schmults, Blitzblau et al. 2023). The primary carcinogen for both BCCs and SCCs is ultraviolet radiation (UVR). UVR is comprised of UVA, UVB, and UVC (which is filtered by the ozone layer); UVR causes direct DNA damage via cyclobutane dimers, 6-4 photoproducts, and DNA double-stranded breaks (Bowden, 2004). UVR mutation signatures have been seen in *p53* , a gene that is mutated at a high frequency in both BCCs and SCCs (Ziegler, 1993). Moreover, *Ha-Ras* has been shown to be mutated at high frequency in NMSC (Pierceall, 1991).

There is a growing body of literature correlating high fat diets to increased tumor growth and higher rates of metastases, suggesting that there is an interplay between lipid metabolism and tumorigenesis. Many reports look at tumorigenesis and lipid metabolism through the lens of feeding model organisms high-fat diets to promote tumorigenesis or by conducting meta-analyses (Kim, Choi et al. 2011, Kyrgiou, 2017, Peck and Schulze, 2019). Thus, we sought to determine if skin cancer cells metabolized triglycerides differently than their normal cell counterparts in a diet-independent model system.

The *in vitro* mouse keratinocyte data for these studies utilized a model that was comprised of three cell lines which represent the progression of non-melanoma skin cancer using the well studied multi-stage skin carcinogenesis protocol of DMBA and TPA (DiGiovanni 1992). A non-transformed cell line that contains wild-type *Ha-Ras* as well as wild-type *p53* was denoted as C5. P1 cells were isolated from the benign papillomas of mice that had been treated with DMBA and the tumor promoting agent TPA. The transformed spindle carcinoma cell line, denoted as A5, was isolated from the tumors of mice that had been subjected to the multi-stage chemical carcinogenesis protocol. In addition to DMBA treatment, these mice were treated with TPA weekly for 40 weeks. A5 cells contain 1 wild-type allele and 2 mutant alleles for *Ha-Ras* at codon 61 as well as a mutated *p53* gene (Zoumpourlis, Solakidi et al. 2003).

To determine whether triglyceride storage was altered in keratinocytes at different stages of tumorigenesis, we measured triglycerides normalized by total protein content in non-transformed keratinocytes (C5), papilloma cells (P1) and squamous carcinoma cells (A5) after treating these cells with oleic acid to promote lipid storage. C5 cells stored significantly less triglycerides than both P1 cells and A5 cells (Figure 1). These data suggest that the storage of triglycerides is promoted in transformed skin cancer cell lines. These results are in agreement with previous studies that show tumor cells have enhanced lipid droplet formation and suggest that the tumor microenvironment favors conditions with higher lipid content. One hypothesis for this is that tumors use the excess lipids for energy and/or to maintain cancer stem cell populations (Li, Condello et al. 2017). Interestingly, a recent study by Liu et. al described a model in which lipid droplets drove cancer progression via MDM2-mediated p53 degradation (Liu, Jing et al. 2025). These studies align with our data as the A5 cells are known to have a mutated *p53* gene. Since we have shown that P1 cells also store excess triglycerides, perhaps a similar cancer driving mechanism is at play in these cells and additional studies are needed to address this outstanding question.

Overall, our studies add to the growing body of literature linking tumorigenesis and lipid homeostasis, describing tumor microenvironments as having increased lipid metabolism.

## Methods

**Tissue Culture**

C5N, P1 and A5 cells (a generous gift from Dr. Allan Balmain) were cultured in DMEM supplemented with 10% fetal bovine serum, 1% penicillin/streptomycin, and 1% glutamine in a 6-well plate (ThermoFisher, Waltham, MA, USA). The cells were treated with 1 mM oleic acid (Sigma, St. Louis, MO, USA) at 60% confluency for 24h. Cells were then washed in 1XPBS (ThermoFisher) and then harvested in 250 μL lysis buffer on ice by cell scraping (140 mM NaCl, 50 mM Tris-HCl, pH 7.4, 0.1% Triton X, and 1X Protease Inhibitor) (Castillo-Quan, Steinbaugh et al. 2023). The cell lysate was then centrifuged at 4°C for 15 min at 16,000Xg. The supernatant was transferred to a clean 1.5 mL microcentrifuge tube for protein and triglyceride analysis. Passages 10-20 were used in these experiments. Stock flasks and experimental plates were incubated at 37°C in a humidified atmosphere of 95% air, 5% CO _2_ .

**Triglyceride assay**

Total protein was measured using the Pierce BCA Protein Assay kit (ThermoFisher) and Triglycerides were measured using the Infinity Triglyceride kit (Fisher Scientific, Pittsburgh, PA, USA) as previously described (Leon et al. 2020). Triglycerides levels were normalized to total protein.

**Statistics**

The results are represented as the mean +/- standard error (SE). Comparisons among the three cell lines were made using a one-way analysis of variance (ANOVA) and post-hoc Tukey test. * denotes p<0.05 and £ denotes p<0.005 (n=48 (P1) or 60 (C5 and A5)).

## Data Availability

Description: The first tab is the pooled accumulated data that the figure is derived from. The colors correspond to individuals who produced the data. The second tab is the data placed in Excel for graphing purposes. The values represent triglycerides/ug protein.. Resource Type: Dataset. DOI: https://doi.org/10.22002/cqk1n-ga648
